# Insulin receptor substrate 1 is a substrate of the Pim protein kinases

**DOI:** 10.18632/oncotarget.7918

**Published:** 2016-03-04

**Authors:** Jin H. Song, Sathish K. R. Padi, Libia A. Luevano, Mark D. Minden, Daniel J. DeAngelo, Gary Hardiman, Lauren E. Ball, Noel A. Warfel, Andrew S. Kraft

**Affiliations:** ^1^ Department of Cellular and Molecular Medicine, University of Arizona, Tucson, AZ 85724, USA; ^2^ University of Arizona Cancer Center, University of Arizona, Tucson, AZ 85724, USA; ^3^ Princess Margaret Cancer Centre, University Health Network, Toronto, ON, M5G 2M9, Canada; ^4^ Department of Medical Oncology, Dana-Farber Cancer Institute, Harvard Medical School, Boston, MA 02215, USA; ^5^ Departments of Medicine and Public Health Sciences and The Center for Genomics Medicine, Medical University of South Carolina, Charleston, SC 29425, USA; ^6^ Department of Cell and Molecular Pharmacology, Medical University of South Carolina, Charleston, SC 29425, USA

**Keywords:** insulin, IGF1, IRS1, Pim kinase, Pim kinase inhibitor

## Abstract

The Pim family of serine/threonine protein kinases (Pim 1, 2, and 3) contribute to cellular transformation by regulating glucose metabolism, protein synthesis, and mitochondrial oxidative phosphorylation. Drugs targeting the Pim protein kinases are being tested in phase I/II clinical trials for the treatment of hematopoietic malignancies. The goal of these studies was to identify Pim substrate(s) that could help define the pathway regulated by these enzymes and potentially serve as a biomarker of Pim activity. To identify novel substrates, bioinformatics analysis was carried out to identify proteins containing a consensus Pim phosphorylation site. This analysis identified the insulin receptor substrate 1 and 2 (IRS1/2) as potential Pim substrates. Experiments were carried out in tissue culture, animals, and human samples from phase I trials to validate this observation and define the biologic readout of this phosphorylation. Our study demonstrates in both malignant and normal cells using either genetic or pharmacological inhibition of the Pim kinases or overexpression of this family of enzymes that human IRS1^S1101^ and IRS2^S1149^ are Pim substrates. In xenograft tumor experiments and in a human phase I clinical trial, a pan-Pim inhibitor administered *in vivo* to animals or humans decreased IRS1^S1101^ phosphorylation in tumor tissues. This phosphorylation was shown to have effects on the half-life of the IRS family of proteins, suggesting a role in insulin or IGF signaling. These results demonstrate that IRS1^S1101^ is a novel substrate for the Pim kinases and provide a novel marker for evaluation of Pim inhibitor therapy.

## INTRODUCTION

Proviral integration site for Moloney murine leukemic virus (Pim) proteins are a family of serine/threonine kinases composed of three different isoforms (Pim1, Pim2 and Pim3) that are aberrantly expressed in a wide variety of tumor types, including hematopoietic malignancies. The oncogenic activity of Pim kinases has been well described in Eμ-myc driven mouse lymphomagenesis, where expression of Pim accelerated Myc driven tumorigenesis [1–3]. It was recently shown that each Pim isoform can collaborate with c-Myc to induce leukemic development [4]. Deletion of all three Pim kinases resulted in abnormalities in mitochondrial metabolism, including a low level of coenzymes NAD^+^/NADP^+^ that led to repression of glycolysis and the pentose phosphate pathway [5]. Given the potential role of Pim in regulating malignant transformation, a number of newly developed pan-Pim kinase inhibitors are currently being tested in clinical trials [6, 7]. For example, the pan-Pim kinase inhibitor AZD1208 [6] entered Phase I trials for acute myeloid leukemia (AML) and solid tumor patients. LGH447, orally available form of LGB321, is being tested in Phase I trial in patients with relapsed and/or refractory multiple myeloma [8, 9] and phase Ib/II trials (CLGH447X2103C; NCT02144038; EudraCT2013-004959-21) are underway targeting for patients with relapsed/refractory AML, or high risk myelodysplastic syndrome as well as patients with myelofibrosis.

Overexpression of Pim kinases in cells exerts a pleiotropic effect on various signal transduction pathways, including cell cycle progression, cell proliferation and apoptosis. To understand how Pim is regulating this diverse set of pathways, it is essential to define the substrates for this protein kinase. Several proteins have been suggested to be regulated by Pim phosphorylation, including cdc25A, BAD, PTP-U2S, PAP-1, p21, NFATc1 and SOCS-1 [10]. Recently, Pim2 was demonstrated to phosphorylate BAD, eIF4B, and apoptosis inhibitor 5 [11, 12]. Pim kinases phosphorylate the AKT phosphorylation consensus, K/RXRXXpS/pT, and multiple common substrates have been identified between these two protein kinases [13]. However, the sequence K/RXRHXpS/pT is phosphorylated 20-fold more efficiently by the Pim protein kinases [11]. This suggests that the scanning protein sequences for the phosphorylation *consensus* K/RXRHXpS/pT could help in identifying potential substrates of Pim protein kinase.

This analysis led to the discovery that IRS1 contains a highly conserved Pim phosphorylation sequence at S1101. Given the role of Pim in regulating a signal transduction pathway related to metabolism [5, 14, 15], this potential substrate was investigated further as a potential biomarker of Pim kinase activity.

## RESULTS

### Pim protein kinases regulate IRS1 phosphorylation

**Figure 1:**
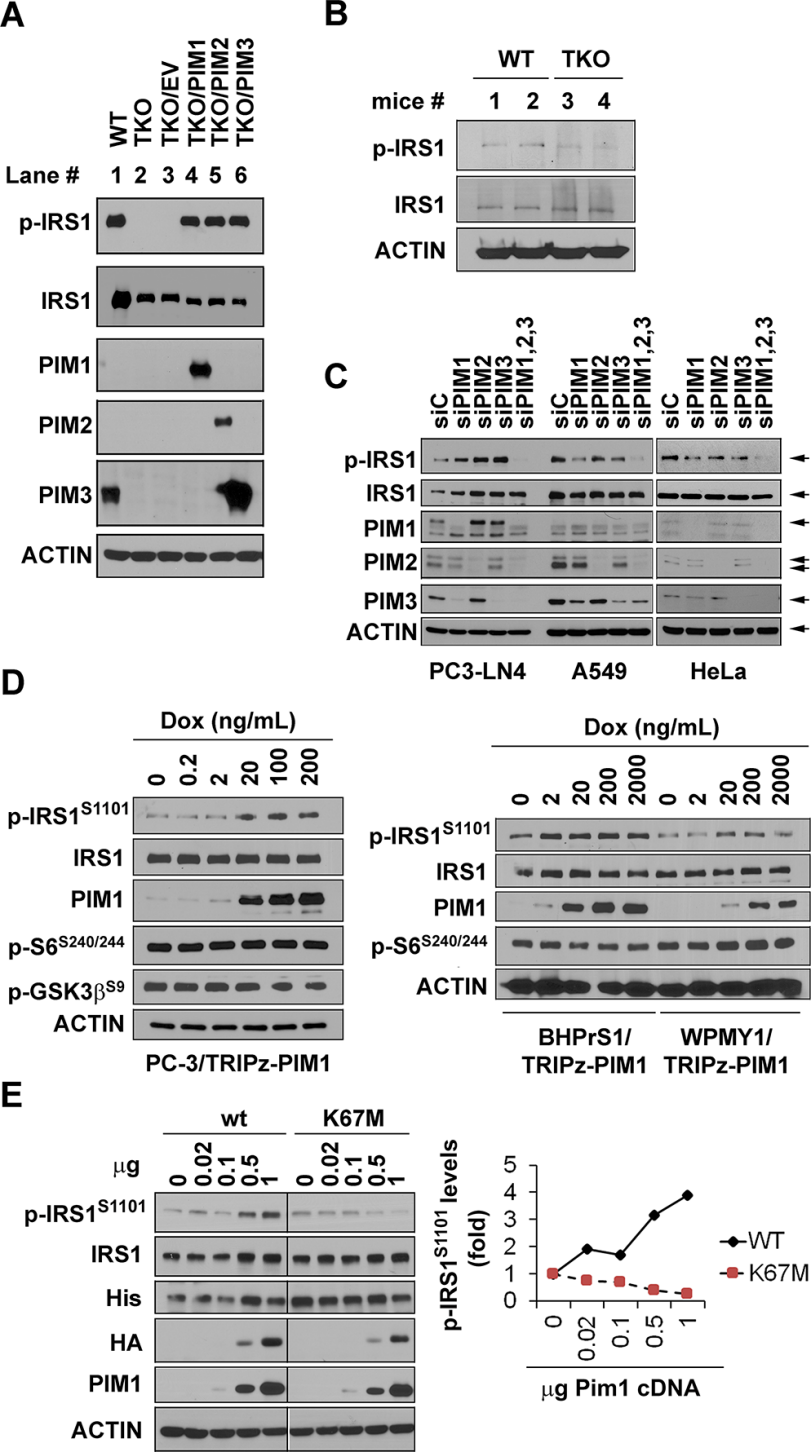
Expression of Pim1, 2, and 3 kinases control IRS1^S1101^ phosphorylation (**A**) IRS1^S1101^ (IRS1^S1097^ in mouse) expression levels in WT, TKO, and TKO MEF cells expressing a single isoform of Pim kinase. (**B**) IRS1^S1101^ (IRS1^S1097^ in mouse) expression levels in kidney tissues of WT and TKO mice (two mice for each). Cell lysates of IRS1 expressing HEK293T transfectants were used as positive controls. (**C**) PC3-LN4, A549 and HeLa cells were transfected with siRNA targeting Pim1, 2, 3 or all three Pims and analyzed after 48 hr. (**D**) Prostate cancer PC-3 cells expressing tet-inducible Pim1, and human prostate fibroblast cell lines BHPrS1 and WPMY1 expressing tet-inducible Pim1 were stimulated with doxycycline at the indicated doses for 48 hr. Western blots were probed with the listed antibodies. (**E**) HA-tagged wild type (wt) and kinase dead mutant (K67M) Pim1 was co-transfected with his-tagged IRS1 in HEK293T cells. Densitometry of Western blot data was shown to measure the levels of Pim phospho-S1101 IRS1. These were normalized to total IRS1.

To search for proteins possessing similar phosphorylation consensus sites, we utilized the NetworKIN resource, a comprehensive database of predicted kinase–substrate relations derived from the human phosphoproteome and integrating interaction networks from the Phospho.ELM, PhosphoSite and STRING databases [14, 16, 17]. The NetworKIN database [18] was queried using AKT and Pim2 kinases for potential substrates. This uncovered 1,247 predicted substrates for Pim2 and 598 for AKT. Among them, 28 proteins contained RXRHXpS/pT Pim phosphorylation recognition motif. This highly conserved consensus sequences was observed on human IRS1 S1101 (S1097 in mouse) and IRS2 S1149 (S1138 in mouse). The context and ranking scores for these target positions were amongst the highest (IRS1 0.983/13.634 and IRS2 0.982/13.62), indicating that IRS1/2 were potential substrates for Pim kinases. To investigate whether IRS is an *in vivo* substrate for Pim protein kinases, MEF cells derived from wild type (WT) and triple knockout of Pim1, Pim2 and Pim3 (TKO) FVB mice were examined. Western blot analysis demonstrated that phosphorylated IRS1 protein expression was undetectable in TKO cells when protein was probed with anti-phospho S1101 IRS1 antibody (Figure 1A; lane 1 and 2). Western blot analysis of kidney tissues from WT and TKO mice also demonstrated that IRS1 phosphorylation was markedly reduced in TKO mouse tissues (Figure 1B). To identify whether one or all of the three PIM isoforms were regulating the phosphorylation of IRS1, each isoform was transduced into TKO cells using lentiviruses producing Pim1, Pim2 or Pim3. Each of the three isoforms was sufficient to induce the phosphorylation of IRS1 on S1101 (Figure 1A; lane 3 to 6). Consistent with these results, the depletion of each Pim kinase isoform individually using siRNA in the prostate cancer cell line PC3-LN4 cells did not decrease IRS1 phosphorylation, but the knockdown of all three isoforms abolished the phosphorylation of the IRS1 protein (Figure 1C). Similarly, depletion of Pim1, 2 and 3 by siRNAs in non-small cell lung carcinoma cell line (A549) and a cervical cancer cell line (HeLa) abolished phosphorylation of IRS proteins on S1101.

The Pim1 protein kinase plays an important role in the initiation and progression of human prostate cancer and is elevated in both epithelial and stromal tumor cells. To determine whether overexpression of Pim1 in prostate cancer increases IRS1 S1101 phosphorylation, human prostate cancer PC-3 cells and human prostate stromal cells, BHPrS1 and WPMY1, expressing tet-inducible Pim1 were studied. The latter cell line was stimulated with varying doses of doxycycline. After 48 hours stimulation, both Pim1 expression and IRS1 phosphorylation increased in parallel to the concentration of doxycycline applied (Figure 1D), suggesting that Pim1 levels regulate the extent of phosphorylation of the IRS1 protein. To further test whether the kinase activity of Pim was needed for this phosphorylation, 293T cells were transfected with increasing amounts of HA-tagged wild type Pim1 wild type or a kinase deficient mutant (*K67M*) Pim1 [19, 20]. Increased expression of wild type Pim1 increased IRS1 phosphorylation (Figure 1E), while the K67M mutant had no effect, demonstrating the importance of the kinase activity of Pim.

### Pim kinases phosphorylate IRS1 and IRS2 proteins

**Figure 2:**
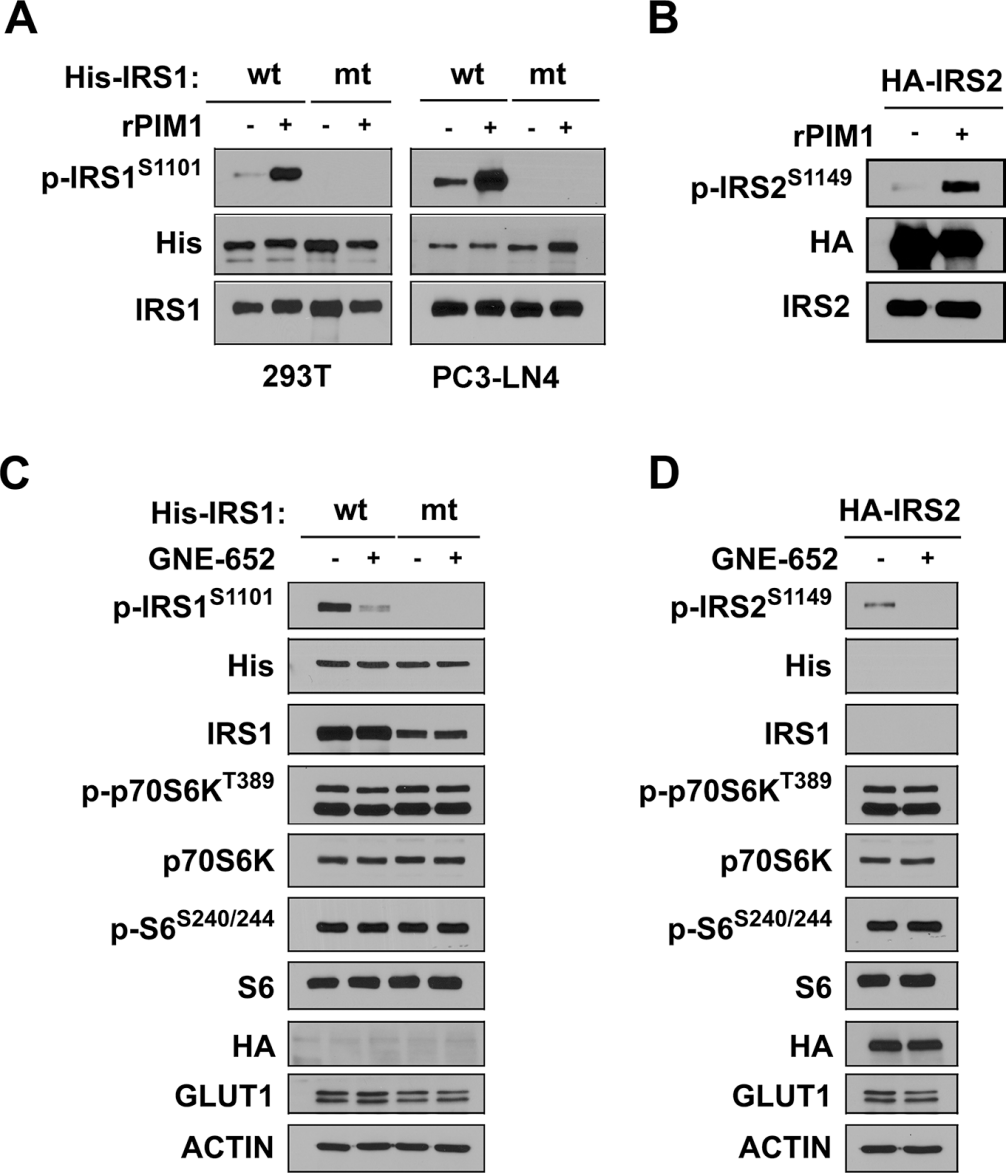
Pim kinase phosphorylates IRS1^S1101^ and IRS2^S1149^ proteins (**A**) Wild type his-tagged IRS1 and its mutant S1101A were transfected into 293T (left) and PC3-LN4 (right) cells followed by immunoprecipitation with anti-His antibody. *In vitro* kinase activity was documented by Western blot analysis of S1101 phosphorylation of IRS1 in His-IRS1 immune complex with or without the addition of recombinant active Pim1 kinase (rPim1). (**B**) Extracts of HA-IRS2 transfected HEK293T cells were immunoprecipitated with anti-HA antibody and subjected to an *in vitro* kinase assay. Addition of recombinant active Pim1 kinase (rPim1) to HA-tagged immune complex induced IRS21^S1149^ phosphorylation. (**C**) LNCaP cells transfected with either wild type (wt) or S1101A mutant (mt) of His-IRS1 for 48 hr were treated with DMSO or the Pim inhibitor GNE-652 (0.2 μmol/L) for 30 min. Extracts were then Western blotted and probed with the antibodies listed. (**D**) LNCaP transfectants expressing HA-IRS2 were treated with DMSO or GNE-652 (0.2 μmol/L) for 30 min and Western blots carried out.

To determine whether Pim1 is capable of directly phosphorylating IRS1 protein on S1101, His-tagged IRS1 was transfected into 293T cells. After 48 hours, His-IRS1 proteins were immunoprecipitated with agarose beads and an anti-His monoclonal antibody. In an *in vitro* kinase assay, the immunoprecipitants were incubated with 0.1 μg of recombinant active Pim1 kinase in the presence of ATP and then subjected to Western blot analysis with anti-phospho S1101 IRS1 antibody. These results demonstrated that recombinant Pim1 could phosphorylate S1101 (Figure 2A). Mutation of this residue in IRS1 from serine to alanine (S1101A) completely abolished *in vitro* phosphorylation by Pim1. Identical results were obtained when His-IRS1 and His-IRS1 S1101A mutant plasmids were transfected into PC3-LN4 prostate cancer cells. Similar results, but less dramatic results, were obtained using the IRS2 protein that contained an identical Pim kinase phosphorylation consensus at 1149 (Figure 2B). To determine that the Pim kinase phosphorylates the IRS protein *in vivo*, LNCaP prostate cancer cells that express only IRS2, and not IRS1, were transfected with His-tagged wild type IRS1 or the S1101A mutant (Figure 2C). In LNCaP cells, the mutant IRS1 was not phosphorylated by Pim1. Treatment with GNE-652, a small molecule pan-Pim inhibitor [5, 7, 21], markedly inhibited the phosphorylation of wild type IRS1. This compound also blocked the phosphorylation of HA-IRS2 when transfected into LNCaP cells (Figure 2D). The inhibition of Pim activity either secondary to a genetic knockout (Figure 1A) or addition of a small molecule inhibitor to cells decreased the level of IRS1 phosphorylation identifying Pim as a protein kinase capable of phosphorylating this site.

### IRS1 S1101/S1097 is not a substrate for the AKT/mTOR/p70S6K pathway

**Figure 3:**
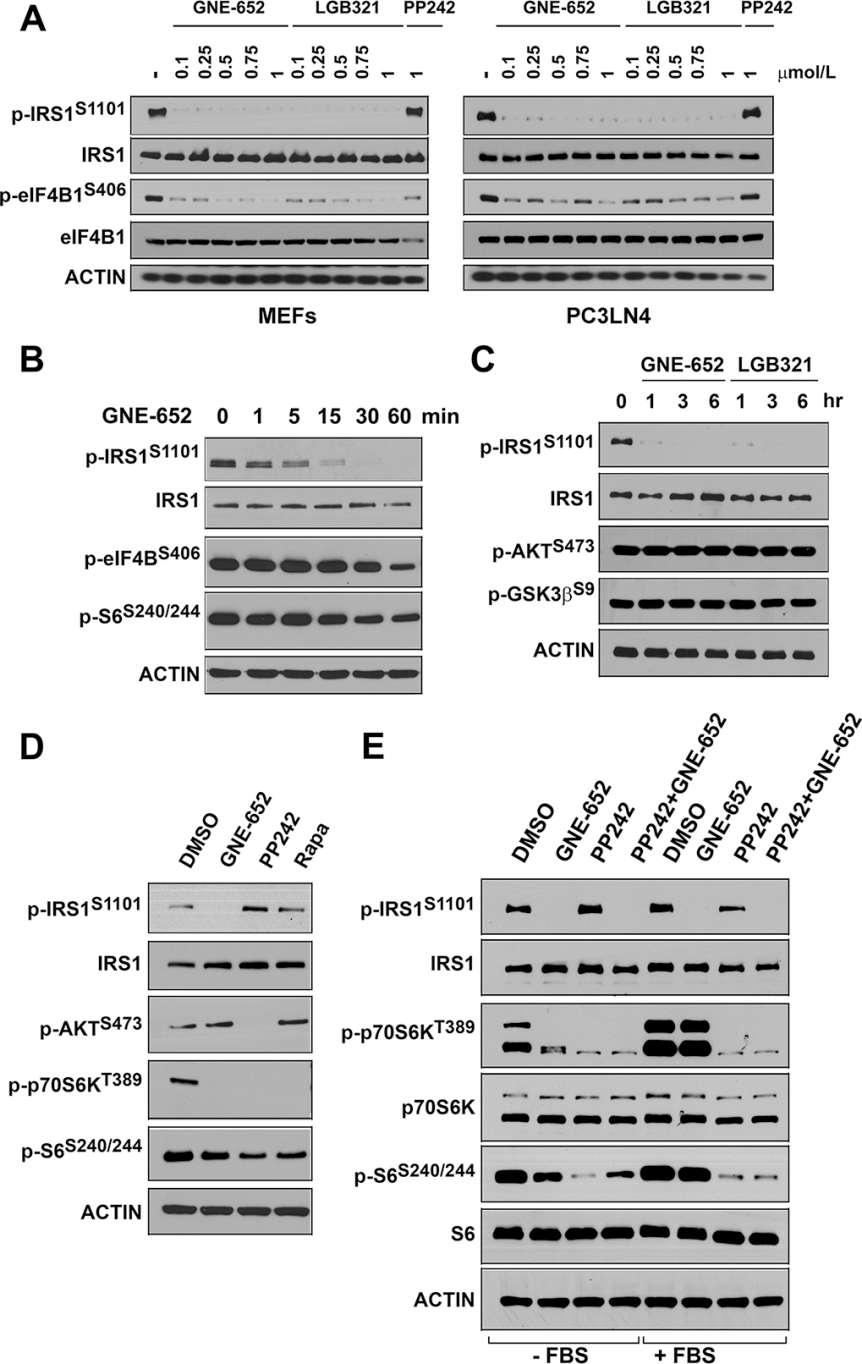
Pim kinase inhibitor blocks IRS1^S1101^ phosphorylation independent of mTOR activity (**A**) MEF and PC3-LN4 cells were treated with GNE-652 or LGB321 in a dose dependent manner and PP242 (1 μmol/L) for 16 hr. (**B**, **C**) PC3LN4 cells were treated with GNE-652 (0.1 μmol/L) or LGB321 (0.1 μmol/L) for various times. (**D**) Calu-1 cells were treated with DMSO, GNE-652 (0.1 μmol/L), PP242 (2 μmol/L), or rapamycin (Rapa, 100 nM) for 1 hr. (**E**) PC3-LN4 cells were serum starved for an hour and then treated with GNE-6652 (0.1 μmol/L), PP242 (2 μmol/L), or GNE-652 plus PP242 for 1 hr. Alternatively, after starvation cells were re-stimulated with 10% FBS together in the presence of inhibitors.

It has been hypothesized [22, 23] that activation of AKT stimulates the mTOR pathway to enhance the activity of p70S6 kinase, which in turn phosphorylates IRS1/2. However, Pim1 overexpression (Figure 1D) did not significantly change the phosphorylation level of ribosomal protein S6 (S240/244), a substrate of activated p70S6K, or phosphorylation of GSK3β (S9) protein, an AKT substrate, suggesting that the AKT/mTOR signaling pathway did not mediate the phosphorylation of IRS1 on S1101. When MEF and PC3-LN4 cells were treated with GNE-652 and LGB321, phosphorylation of S1101/S1097 on IRS1 was abolished at concentrations as low as 100 nmol/L. These compounds inhibited the phosphorylation of eIF4B, a known substrate of the Pim kinase. In contrast, PP242, a known inhibitor of mTOR, did not alter S1101 phosphorylation (Figure 3A and 3B). Reduction of S1101 phosphorylation by the Pim inhibitor was apparent after 15 and 30 min treatment periods (Figure 3B) and was completely abolished after longer treatment with Pim inhibitors (Figure 3C). When the lung cancer cell line, Calu-1, was treated with GNE-652, PP242 or rapamycin, phosphorylation levels of p70S6K and ribosomal protein S6 proteins were all reduced (Figure 3D). PP242, functioning as an mTORC2 inhibitor, decreased the phosphorylation of AKT at S473. However, only treatment with GNE-652 abolished S1101 phosphorylation. When serum starved PC3-LN4 cells were stimulated with serum, phosphorylation of p70S6K and ribosomal protein S6 proteins were markedly increased (Figure 3E). GNE-652 treatment reduced the phosphorylation of p70S6K and S6 in serum starved cells, but not in serum treated cells, suggesting that Pim plays a role in mTOR regulation in the absence of growth factors. In contrast, PP242 reduced the phosphorylation of these two proteins in the absence and presence of serum. In comparison to the regulation of mTOR, IRS1 S1101 phosphorylation was only significantly decreased by the addition of Pim kinase inhibitors.

### Pim kinase inhibitor prevents IRS1 S1101 phosphorylation under exposure of IGF1 and insulin

**Figure 4:**
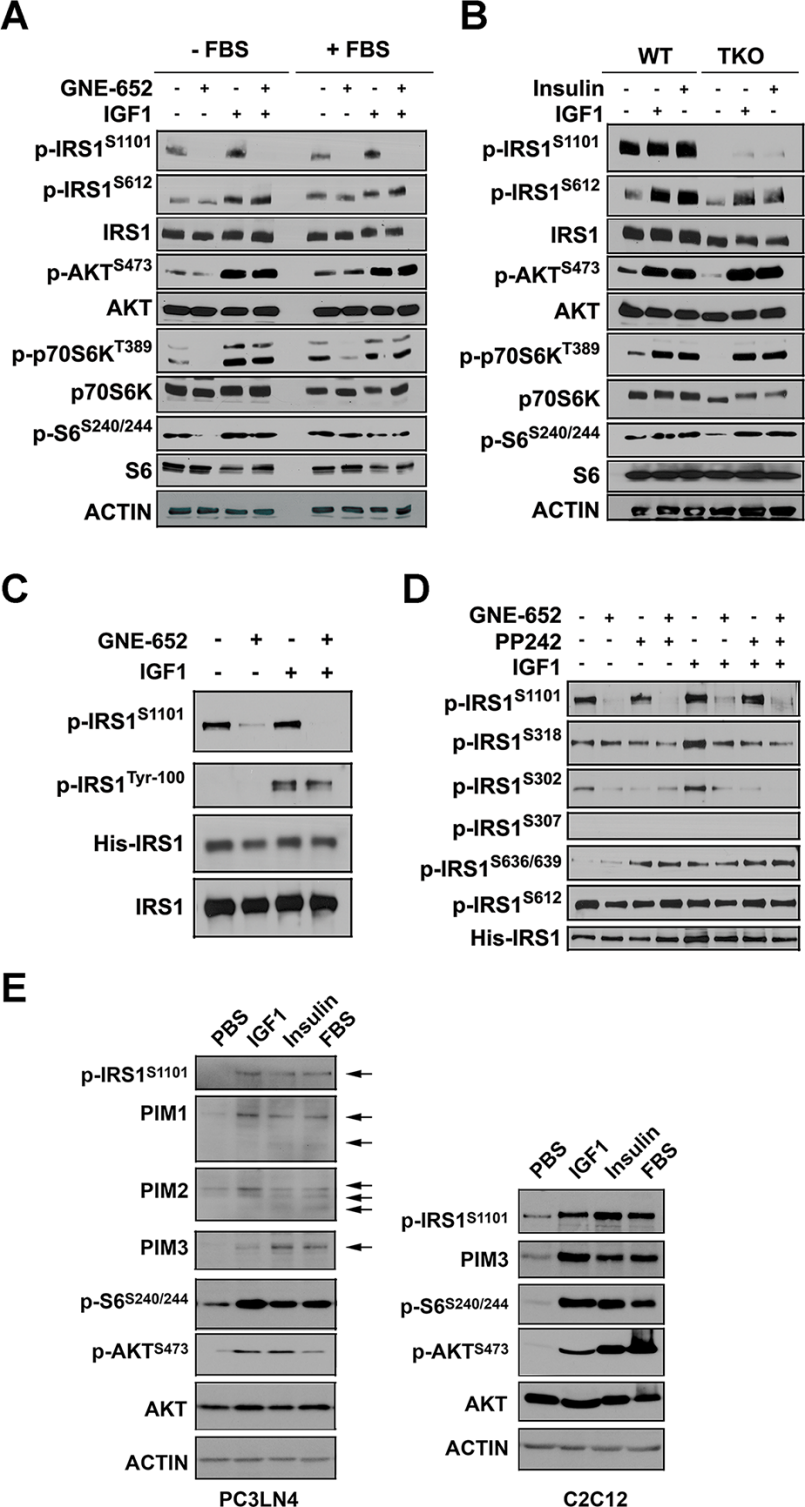
Inhibition of Pim kinases abrogates IRS1^S1101^ phosphorylation after insulin administration (**A**) PC3-LN4 cells were stimulated with IGF1 (100 ng/mL) for 15 min. Prior to stimulation, cells were treated with DMSO or GNE-652 for 1 hr in the absence or presence of FBS. Extracts of the cells were made and Western blots carried out as shown. (**B**) WT and TKO MEF cells were serum starved for 1 hr and then stimulated with IGF1 (100 ng/mL) or insulin (400 ng/mL) for 15 min. Western blots were carried out with the labeled antibodies. (**C**) Western blot analysis of His-IRS1 immunoprecipitation samples. (**D**) Effects of GNE-652 on serine residues of IRS1. HEK293T transfectants expressing IRS1 were treated with either GNE-652 (100 nmol/L) or PP242 (2 μmol/L) for 1 hr and then exposed to IGF1 for 15 min. Cells were lysed and the resulting lysates were incubated with anti-His antibody and A/G agarose beads. Immunoprecipitated samples were subjected to Western blot analysis. (**E**) Effects of growth factors on Pim kinase protein expression. PC3-LN4 and C2C12 cells were serum-starved for 16 hr and then exposed to PBS, IGF1 (100 ng/mL), insulin (400 ng/mL) or 10% FBS for 6 hr. Lysed samples were subjected to Western blot analysis.

IRS1 is phosphorylated in response to the addition of insulin or IGF1 to cells. Serine/threonine phosphorylation of IRS1 has been demonstrated to be a negative regulator of insulin signaling, and it is responsible for the degradation of IRS1. Serine 612 is reported to be one of the sites that can be phosphorylated in response to insulin treatment [24]; S1101 has not been well investigated. Western blots demonstrate that IGF1 treatment of starved cells increased IRS1 phosphorylation at both S1101 and S612. S1101 phosphorylation was abolished by GNE-652 treatment while this pan-Pim inhibitor had no effect on S612 phosphorylation (Figure 4A). It was previously demonstrated that p70S6K is capable of phosphorylating the 612 site [24]. Western blots demonstrate the hormonal activation of AKT, p70S6K, and pS6 phosphorylation was not inhibited by GNE-652. In both WT and TKO MEF cells, both IGF1 and insulin treatment increased S612 phosphorylation, as well as stimulating the AKT/p70S6K pathway (Figure 4B), but in contrast, S1101/S1097 phosphorylation was not phosphorylated in TKO cells.

To examine whether IGF1-mediated phosphorylation at other residues was altered by Pim inhibition, 293T cells were transfected with His-IRS1 and then treated with the Pim inhibitor, GNE-652. His-tagged IRS1 immunoprecipitants were analyzed by Western blotting with anti-phospho S1101, S318, S302, S307, S612, and tyrosine (P-100) antibodies. Phosphorylation at IRS1 on tyrosine residues was increased after IGF1 stimulation but it was unchanged by GNE-652 treatment (Figure 4C and 4D). In contrast, S1101 and S302 phosphorylation was markedly reduced by GNE-652 treatment. PP242, an mTOR inhibitor, also decreased S302 but not S1101, suggesting that the ability of Pim to regulate the mTOR pathway may explain the regulation of S302. Other serine residues, including S318, S612 and S636/369, were not markedly altered by GNE-652; S307 phosphorylation was not detected. We further quantitated human IRS1 phosphopeptides by LC-MS/MS analysis of His-tagged IRS1 immunoprecipitants. Experiments were carried out after IGF1 stimulation in the presence or absence of Pim kinase inhibitor. Using this technique, Pim inhibitor treatment was again shown to completely prevent IGF1 stimulated phosphorylation of S1101 on human IRS1 protein (data not shown). Additional Western blot analysis demonstrated the ability of insulin, IGF1 or serum stimulation to increase expression of Pim kinases in serum starved PC3LN4 and C2C12 cells (Figure 4E) possibly accounting for the increased phosphorylation of IRS proteins. This data suggests that IGF1 and insulin modulate the activity of Pim kinases to phosphorylate IRS1. The addition of Pim inhibitors in serum starvation may regulate the mTOR pathway and affect additional IRS1 phosphorylation sites.

### The Pim kinases mediate PKC-directed phosphorylation of S1101

**Figure 5:**
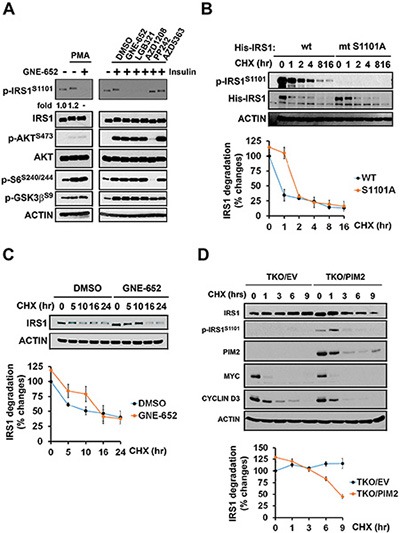
PMA stimulation of IRS1 phosphorylation is mediated by the Pim kinase IRS1^S1101^ phosphorylation minimally influences on IRS1 protein turnover. (**A**) C2C12 cells were stimulated with PMA in presence or absence of GNE-652 for 3 hr (fold changes in phosphorylation of IRS1 in the presence of PMA are shown by densitometry), or pretreated with DMSO, GNE-652 (100 nmol/L), LGB321 (100 nmol/L), AZD1208 (100 nmol/L), PP242 (2 μmol/L), or AZD5363 (2 μmol/L) and then stimulated with insulin for 15 min. (**B**) C2C12 transfectants expressing wild type and S1101A mutant His-IRS1 were treated with cycloheximide (100 μg/mL) for 0, 5, 10, 16, or 24 hr for His-tagged IRS1 protein degradation (an example of the experiment repeated in duplicate). (**C**) Endogenous IRS1 protein turn over in PC3-LN4 cells treated with the Pim kinase inhibitor GNE-652 (0.2 μmol/L). (**D**) TKO MEF cells expressing Pim 2 or empty vector (EV) were treated with cycloheximide (100 μg/mL) for 0, 1, 3, 6, or 9 hr to inhibit protein synthesis and allow the measurement of the rate of IRS1 protein degradation.

IRS1^S1101^ is reportedly phosphorylated by protein kinase C (PKC) [25], which requires diacylglycerol for its kinase activity [26, 27]. Phorbol ester, 12-myristate 13-acetate (PMA), treatment of cells activates PKC [28, 29] and NF-kB [30]. The addition of PMA to C2C12 myoblast cells has been shown to stimulate IRS1^S1101^ phosphorylation. In our experiments PMA appeared to increase phosphorylation by 20%, and this phosphorylation was inhibited by GNE-652 (Figure 5A). In contrast to inhibitors of Pim protein kinases, PP242, a mTOR inhibitor and AZD5363, an AKT inhibitor did not cause a change in the phosphorylation of IRS1^S1101^. In these cells, the Pim inhibitors did not alter PKC kinase activity (data not shown). These results indicate that the Pim protein kinases are the primary regulator of IRS1^S1101^ phosphorylation, and suggest that these enzymes might function downstream of PKC in the control of the phosphorylation of this site.

### Inhibition of Pim kinases delays IRS1 degradation

Phosphorylation at serine/threonine residues on IRS1 has been suggested to be a mechanism of insulin resistance by controlling IRS1 degradation and thus the ability of insulin to signal. To assess whether inhibition of S1101 phosphorylation induces a change in IRS1 protein turnover, C2C12 cells expressing IRS1 wild-type and S1101A mutant cells were treated with cycloheximide. Compared to wild-type IRS1, His-tagged protein S1101A mutant IRS1 degradation was slightly delayed (Figure 5B). The Pim inhibitor treatment of PC3-LN4 cells also caused a delay in the degradation of endogenous IRS1 (Figure 5C). In contrast to IRS1^S1101^ phosphorylation, Pim inhibitors did not alter Cyclin D3 or c-Myc protein turnover. To further examine whether Pim expression alters IRS1 protein stability, cycloheximide chase was performed in TKO MEF cells expressing Pim2 only or empty vector. As Pim2 expression leads to phosphorylation of S1101, a half-life of IRS1 protein was significantly shortened (Figure 5D). Another indication of the ability of Pim to control IRS1 protein levels is the observation that increases in each of three Pim isoforms in TKO MEFs lowered the basal levels of IRS1 expression (Figure 1A).

### IRS1 S1101 phosphorylation is decreased in both mice and humans treated with Pim inhibitors

**Figure 6:**
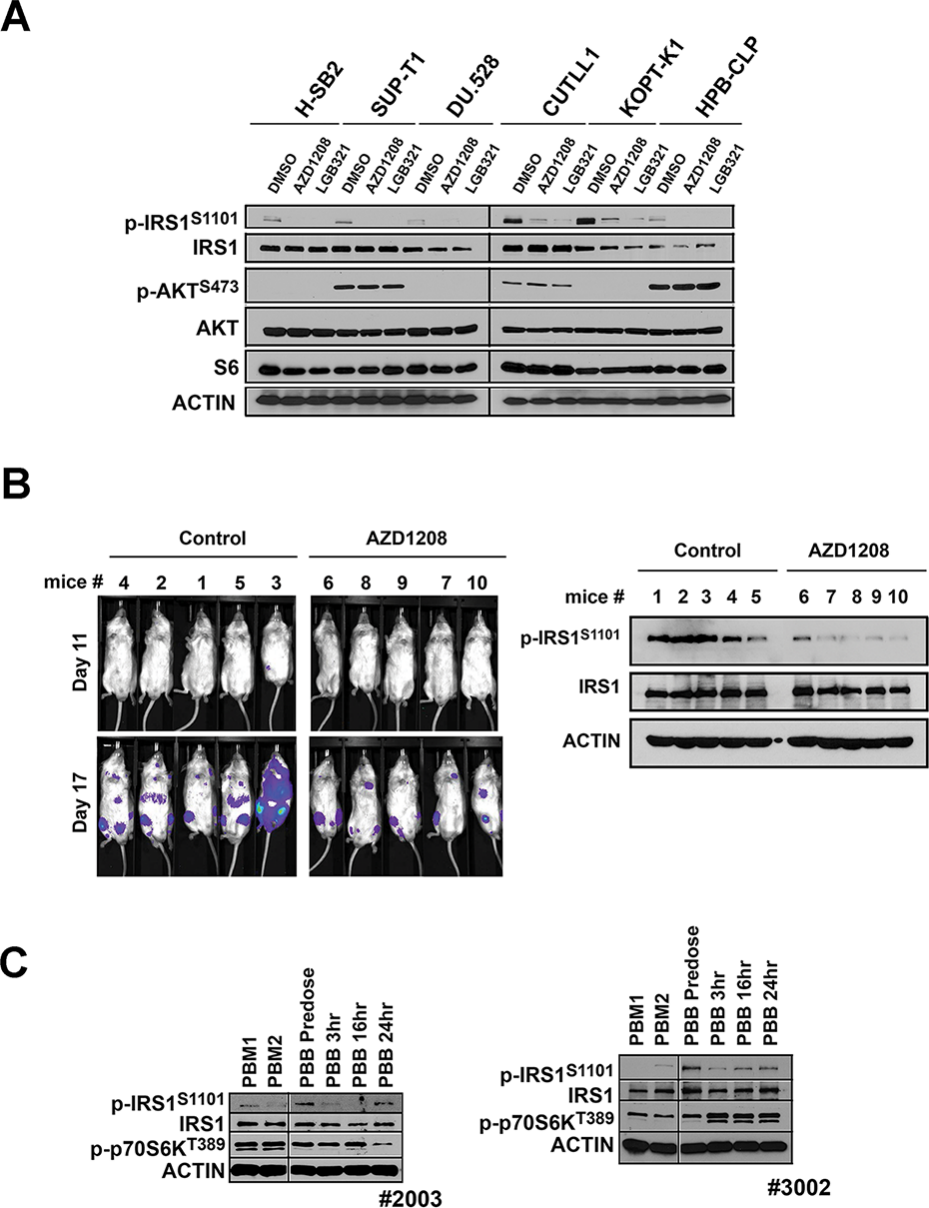
Phosphorylation of IRS1^S1101^ in response to administration of AZD1208, a Pim kinase inhibitor in tumor xenografts and AML patients (**A**) Pim inhibitor AZD1208 reduces IRS1^S1101^ expression in leukemic cells. T-ALL cell lines H-SB2, SUP-T1, DU.528, CUTLL1, KOPT-K1 and HPB-CLP were treated with DMSO, AZD1208 or LGB321 for 24 hr and analyzed by Western blot. (**B**) The luciferase-expressing H-SB2 cells were injected via tail vein into sublethally irradiated NSG mice. Two days after the tumor injection, the mice were treated with AZD1208 (30 mg/kg twice a day by oral gavage) or vehicle control. Tumor growth was monitored by bioluminescence imaging at 11 and 17 days post cell injection. IRS1^S1101^ expression levels were examined by Western Blot analysis in lysates of bone marrow cells at necropsy. (**C**) Pim inhibitor AZD1208 reduces IRS1^S1101^ expression in samples from patients with AML. Analysis of IRS1 S1101 expression in bone marrow (PBM) and peripheral blood monolayer cell extracts (PBB) obtained from patients with AML who underwent AZD1208 therapy in Phase I clinical trial. PBM1 samples are collected at screening (pre dose bone marrow) and PBM2 are collected 2–6 hr after the first dose of AZD1208 (post dose bone marrow). Pre dose peripheral blood and post dose peripheral blood (3, 6, and 24 hr) were collected on day 1 (PBB1) and day 14 (PBB2).

We have previously demonstrated that T-ALL cells show moderate inhibition of growth when treated with Pim inhibitors [31]. Treatment of these human leukemic cell lines in culture with two different Pim inhibitors AZD1208 and LGB321 markedly reduced IRS1^S1101^ phosphorylation (Figure 6A). To examine the ability of Pim inhibitors to function *in vivo* to inhibit tumor growth and regulate IRS1 phosphorylation, we injected NSG mice with H-SB2, T-ALL cells expressing luciferase and administered AZD1208 by oral gavage. Bioluminescence imaging on day 11 after injection did not disclose tumor growth, while by Day 17 treatment with AZD1208 moderately inhibited the growth of these T-ALL cells (Figure 6B). The total bioluminescence differed significantly (*P* = 0.016) between the treated and control mice. After sacrifice, the cells were removed from the mouse bone marrow, and IRS1^S1101^ expression was measured. The cellular extracts from the treated mice demonstrated that AZD1208 reduced IRS1^S1101^ phosphorylation, while inhibiting leukemic cell growth *in vivo*.

To test whether this decrease in IRS phosphorylation also occurred in leukemia patients, samples from a phase I trial of AZD1208 in humans were evaluated. These samples consisted of both human bone marrow and peripheral blood. Inhibition of S1101 after AZD1208 treatment was detected in bone marrow samples (PBM) isolated from patients 3002 (Figure 6C), while patient 2003 appeared to show the reverse effect. Analysis of cell lysates isolated from peripheral blood mononuclear cells detected the reduction of IRS1 S1101 expression after AZD treatment in these patients. Thus, in a limited number of patients treated with a Pim inhibitor *in vivo* measurable changes in IRS1 phosphorylation could be observed. Additional patient samples from solid and liquid tumors will be needed to examine in detail this protein phosphorylation site as a biomarker for Pim inhibitor activity.

## DISCUSSION

Using a bioinformatics approach, the IRS1 and 2 proteins have been identified as potential substrates for the Pim kinases. Experiments carried out both *in vitro*, using immunoprecipitated proteins, and *in vivo*, with siRNA to the three Pim kinases and pan-Pim inhibitory drugs, demonstrated that Pim is capable of phosphorylating in humans IRS1^S1101^ and IRS2^S1149^. Phosphorylation of these sites was absent in MEFs from mice that do not contain any of the Pim kinase isoforms (TKO), validating the hypothesis that IRS1 and 2 are targets for the Pim kinases. In human prostate cancer cells, it was necessary to knockdown all three Pim kinases to inhibit phosphorylation of IRS proteins. Overexpression of a single Pim isoform in TKO was sufficient to induce IRS phosphorylation, suggesting that each of these enzyme can phosphorylate the IRS proteins and they have overlapping biologic activities. Likewise, each of the Pim kinases is capable of collaborating with Myc to enhance transformation [1, 4]. Previously, we identified an identical consensus sequence in eIF4B^S406^ [21], and this protein was also shown to be an excellent Pim kinase substrate. Although additional substrates have not been examined, this bioinformatics approach, examining highly conserved Pim phosphorylation sequences, is likely to yield a more complete knowledge of the substrates phosphorylated by this enzyme.

Previous experiments carried out by others [25, 32] suggested a role for both p70S6 kinase and PKC in regulating the phosphorylation of IRS1. p70S6K has been shown to phosphorylate serine residues on IRS1, including S307 [33, 34], S312, S616, S636/S639 [35] and S1101 [32]. However, both rapamycin and PP242, inhibitors of mTORC1/2, blocked phosphorylation p70S6K and AKT, but did not significantly reduce IRS1^S1101^ expression. This observation is consistent with a previous report showing that S1101 phosphorylation was absent in TSC1/2-null cells where p70S6K and AKT kinases are highly activated [36]. The phosphorylation IRS1^S1101^ was previously suggested to be regulated by PKC*θ* [25]. In our experiments, a small increase in IRS1^S1101^ phosphorylation was observed after treatment of myoblasts with the PKC activator, PMA, and this increase was inhibited by the addition of a small molecule Pim inhibitor. It is possible that PMA increased Pim levels through its ability to regulate NF-κB, which then increased the phosphorylation of IRS1. Alternatively, PKC or a downstream kinase activated by PMA could be stimulating Pim activity [37]. Additional experiments are necessary to decipher the role of PKC in the Pim pathway. These results demonstrate that the Pim kinases are the major, if not only, IRS^S1101^ phosphorylating kinase.

In multiple cell types, structurally different Pim kinase inhibitors blocked the ability of insulin to stimulate the phosphorylation of IRS11^S1101^, suggesting that the insulin stimulatory pathway regulates Pim activity. The Pim and Akt pathways appear to be regulated in parallel, as evidenced by their ability to modulate protein synthesis through the regulation of TSC2 and eIF4B [21, 38, 39]. Recent evidence [5] suggests that Pim can modulate cellular metabolism, including glycolysis and mitochondrial energy production. This demonstration and the role of Pim in regulating IRS phosphorylation suggest that Pim and Akt might modulate cellular metabolism in parallel or overlapping pathways. It has been suggested that IRS1^S1101^ phosphorylation contributes to a decrease in the essential tyrosine phosphorylation needed for IRS1 function [32]. However, complete inhibition of Pim did not alter the ability of IGF1 or insulin to fully activate the tyrosine phosphorylation of IRS proteins. Serine phosphorylation is thought to significantly decrease the half-life of IRS proteins and cause insulin resistance. The data presented here demonstrate that Pim inhibitors or the mutation of S1101 causes small and repeatable changes in IRS1 half-life, but not to the extent reported previously for this site. The fact that IRS1 is phosphorylated on multiple sites, including S307, 312, 612, 636/639, suggests that control of the half-life of this protein may depend on multiple kinase pathways. Thus, blocking insulin resistance by inhibiting IRS1 phosphorylation likely will require the inhibition of multiple enzymes.

Administration of Pim kinase inhibitor AZD1208 to mice bearing tumors blocked S1101 phosphorylation of IRS1 in tumors extracts. Additionally in 2 patient samples that we were able to obtain from patients treated with AZD1208 in a phase I trial for myeloid leukemia, Pim inhibition blocked IRS1^S1101^ phosphorylation. This data points to the possibility that IRS1 phosphorylation could be used as a biomarker of Pim activation or inhibition in clinical trials of anticancer agents targeting this protein kinase.

## MATERIALS AND METHODS

### Antibodies and reagents

The following antibodies were purchased from Cell Signaling Technology: anti-phospho-tyrosine (p-Tyr-100), anti-Pim1, anti-Pim2, anti-Pim3, anti-Cyclin D3, anti-phospho-eIF4B, anti-AKT, anti-phospho-AKT (S473), anti-phospho-AKT (T308), anti-p70S6K, anti-phospho-p70S6K (T389), anti-phospho-S6 (S240/244), anti-phospho-GSK3β (S9), and anti-c-Myc, and anti-phospho IRS1 (S1101, S318, S302, S307, S636/639, S612) antibodies which recognize the following residues in human (h) or mouse (m) IRS1; anti-phospho IRS1 hS1101 (m1097); mS318 (hS323); mS302 (hS307); mS307 (hS312); hS636/S639 (mS632/635); mS612 (hS616). This numbering refers to human IRS1 (P35568); and mouse IRS1 (P35569). Anti-IRS1 (R & D systems), anti-RPS6 (Santa Cruz Biotechnology), anti-His (GenScript), anti-HA and anti-β-actin (Sigma) antibodies were used in these studies. Horseradish peroxidase (HRP)-linked enhanced chemiluminescence (ECL), mouse IgG and rabbit IgG secondary antibodies were purchased from GE Healthcare Life Sciences. Various protein kinase inhibitors, GNE-652 (Genentech), LGB321 (Novartis), AZD1208, AZD5363 (AstraZeneca) and PP242 (Selleck Chemicals) were used in these studies. All other chemicals including cycloheximide (CHX) and PMA were purchased from Sigma. Human recombinant insulin and insulin-like growth factor 1 (IGF1) were obtained from Peprotech.

### Cell culture

PC-3, LNCaP, A549, Calu-1, HEK-293T, HeLa and C2C12 cells were purchased from the American Type Culture Collection (authenticated by short tandem repeats single nucleotide polymorphism, and fingerprint analyses) and were not passaged or maintained for more than 6 months. PC3-LN4 cells were described before [40] and human prostate fibroblast cell lines BHPrS1 and WPMY1 expressing tet-inducible Pim1 [41] were cultured in RPMI supplemented with 2 mmol/L Glutamax (Life Technologies) and 10% fetal bovine serum (BioAbChem) at 37°C under 5% CO_2_ as reported previously. HeLa and C2C12 cells were grown in Dulbecco's modified Eagle medium (DMEM) supplemented with 2 mmol/L Glutamax and 10% fetal bovine serum at 37°C under 5% CO_2_. T-lineage acute lymphoblastic leukemia (T-ALL) cell lines including H-SB2, SUP-T1, DU.528, CUTLL1, KOPT-K1 and HPB-CLP were a gift of Dr. Jon C. Aster (Brigham and Women's Hospital, Harvard University) and cultured in RPMI supplemented with 2 mmol/L Glutamax and 10% fetal bovine serum. Wild-type (WT), triple-knockout (TKO), Pim1^−/−^, Pim2^−/−^, and Pim3^−/−^ mouse embryonic fibroblasts (MEFs) and TKO MEFs expressing Pim1, Pim2 or Pim3 have been described elsewhere [5]. All cell lines were tested for Mycoplasma.

### Plasmids and siRNAs

The his-tagged IRS1 [42] and HA-tagged IRS2 [43] constructs were described previously. Human IRS1 S1101A mutant was generated using the oligonucleotide primers, sense 5′-GCCGGCGGA GGCATAGCGCCGAGACTTTCTCCTC-3′ and antisense 3′-CGGCCGCCTCCGTA TCGCGGCTCTGAAAGAG GAG-5′, and QuikChange^®^ Lightning Site-Directed Mutagenesis Kit according to the manufacturer's protocol. The HA-Pim1 construct was obtained by subcloning human Pim1 cDNA into pLEX vector (Open Biosystems), as described elsewhere [44]. These plasmids were transfected with Xfect transfection reagent (Clontech) according to the manufacturer's instructions. Small interfering RNAs (siRNAs) targeting Pim1, Pim2 and Pim3 (Dharmacon) were transfected with Lipofectamine 3000 reagent (Life Technologies) according to the manufacturer's instructions. To knockdown all three Pim isoforms, siRNAs targeting Pim1, Pim2 and Pim3 were combined (100 pmol). The sequence of each of the siRNAs are as follows: Pim1 sense 5′-GAUAUGGUGUGUGUGGAUA-3′, Pim1 antisense 5′-UAUCUCCACACACCAUAUC-3′, Pim2 sense 5′-ACCUUCUUCCCGACCCUCA-3′, Pim2 antisense 5′- UGAGGGUCGGGAAGAAGGU-3′, Pim3 sense 5′-GCACGUGGUGAAGGAGCGG-3′, Pim3 antisense 5′-CCGCUCCUUCACCACGUGC-3′, and scrambled sequence of nonsilencing control siRNA oligonucleotides were sense 5′-UUCUCCGAACGUGUCACGU-3′ and antisense sequence 5′- ACGUGACACGUUCGGAGAA-3′.

### PCR assay

Reverse transcriptase PCR and quantitative real time PCR (qPCR) assay was performed as previously described [5]. The following primers were used:

mouse IRS1-forward, 5′-CTC AGT CCC AAC CAT AAC CAG-3′; mouse IRS1-reverse, 5′-TCC AAA GGG CAC CGT ATT G-3′; mouse β-Actin-forward, 5′-GAC ATG GAG AAG ATC TGG CA-3′; mouse β-Actin-reverse, 5′-GGT CTC AAA CAT GAT CTG GGT-3′.

### Immunoblotting and immunoprecipitation

Cells were harvested in lysis buffer consisting of 50 mmol/L Tris, pH 7.4, 150 mmol/L NaCl, 1% NP-40, 5 mmol/L EDTA and 1% protease/phosphatase inhibitor cocktail. Following 15 min of incubation in lysis buffer at 4°C, lysates were cleared by centrifugation at 15,000 rpm for 15 min at 4°C, and then protein concentrations were determined by the DC protein assay (Bio-Rad Laboratories). His-tagged IRS1 or HA-tagged IRS2 was immunoprecipitated in a lysis buffer with anti-His or anti-HA antibodies and protein A/G-agarose (Pierce). Densitometry was determined with Image Studio Lite Ver 5.0 (LI-COR Biosciences) with normalization to the corresponding controls.

### IRS1 S1101 *in vitro* kinase assay

His-IRS1, its mutant (S1101A), or HA-IRS2 transfectants were immunoprecipitated with anti-His or anti-HA antibodies. Immune complexes were washed three times in lysis buffer, then washed twice in 1 × kinase buffer (20 mM MOPS [pH 7.0] containing 100 mM NaCl, 10 mmol/L MgCl_2_, and 2 mmol/L dithiothreitol) and then incubated with 0.1 μg of recombinant active Pim1 kinase [44] and 100 μmol/L of ATP for 15 min at 25°C. Reactions were stopped by washing twice in a cold kinase buffer and boiling in 2 × SDS loading buffer. The sample was separated on a SDS-polyacrylamide gel and subjected to Western blot analysis with anti-phospho IRS1 antibodies.

### Bioinformatics analysis of Pim kinase substrates

The NetworKIN resource Version_2_0 (http://networkin.info) was utilized to uncover Pim kinase substrates. NetworKIN is a database of predicted kinase-substrate relationships and the underlying computational approach combines predictions of kinase families expected to phosphorylate particular motifs with background information on substrates and kinases to forecast which kinases mediate phosphorylation at specific sites. The method integrates four sources of evidence, a genomic context, experimental evidence (protein interactions and co-expression), pathway databases and literature mining. A context score is provided to help eliminate false positive results based on consensus motifs alone [45].

### Mouse tissues and tumor xenografted mice

All animal studies were performed in compliance with institutional guidelines under an IACUC approved protocol. To analyze IRS1 S1101 (S1097 in mouse) phosphorylation levels in various tissues including kidney, liver, abdominal fat and muscle tissues were obtained from WT and TKO FVB mice. Tissue extracts were prepared by homogenizing the tissues in RIPA lysis buffer, as described previously [46]. To examine the effects of a Pim kinase inhibitor on IRS1 S1101 phosphorylation levels in *in vivo* tumors, mouse xenograft model of T-cell leukemia, H-SB2 cells stably expressing luciferase (2 × 10^5^ cells/mouse) were injected via tail vein into irradiated (2.5 Gy) 4–6 week old NOD/SCID male mice (Charles River Laboratories). Starting on day 3, the mice were treated with AZD1208 (30 mg/kg twice daily by oral gavage) or control vehicle (0.5% HPMC, 0.1% Tween-80) daily for ~17 days. Tumor growth was followed by bioluminescence imaging at the time-points indicated.

### Clinical samples

Lysates of bone marrow and peripheral blood mononuclear cells of AZD1208 phase I clinical trial patients were kindly provided by AstraZeneca and used as reported previously [21].

## Abbreviations

The abbreviations used are:

CHX: cycloheximide
IGF1: insulin-like growth factor 1
IRS: insulin receptor substrate
mTOR: mammalian target of rapamycin
MEFs: mouse embryonic fibroblasts
Pim: Proviral integration site for moloney murine leukemic virus
PMA: Phorbol ester 12-myristate 13-acetate
qPCR: quantitative real time PCR
TKO: triple-knockout
WT: wild-type
